# Timing of Diapause Initiation and Overwintering Conditions Alter Gene Expression Profiles in *Megachile rotundata*

**DOI:** 10.3389/fphys.2022.844820

**Published:** 2022-03-08

**Authors:** Lizzette D. Cambron-Kopco, George D. Yocum, Kathleen M. Yeater, Kendra J. Greenlee

**Affiliations:** ^1^Greenlee Laboratory, Department of Biological Sciences, North Dakota State University, Fargo, ND, United States; ^2^Insect Genetics and Biochemistry Research Unit, Edward T. Schaefer Agricultural Research Center, USDA-ARS, Fargo, ND, United States; ^3^Plains Area Office of The Area Director, USDA-ARS, Fort Collins, CO, United States

**Keywords:** solitary bee, diapause, overwintering, gene expression, insulin signaling pathway, seasonal variation, *Megachile rotundata*

## Abstract

Within the United States and Canada, the primary pollinator of alfalfa is the alfalfa leafcutting bee (ALCB), *Megachile rotundata*. Our previous findings showed that overwintering conditions impacted gene expression profile in ALCB prepupae that entered diapause early in the season. However, ALCB are a bivoltine species, which begs the question of whether bees entering diapause later in the season also show this trend. To better understand the effects of the timing of diapause initiation, we analyzed mRNA copy number of genes known to be involved in diapause regulation in early and late season diapausing ALCB that were overwintered in field conditions or using current agricultural management conditions. We hypothesized that overwintering conditions for late diapausing bees also affects gene expression profiles. Our results showed that expression profiles were altered by both overwintering condition and timing of diapause initiation, with bees that entered diapause earlier in the season showing different expression patterns than those that entered diapause later in the season. This trend was seen in expression of members of the cyclin family and several targets of the insulin signaling pathway, including forkhead box protein O (FOXO), which is known to be important for diapause regulation and stress responses. But, of the genes screened, the proto-oncogene, *Myc*, was the most impacted by the timing of diapause initiation. Under field conditions, there were significant differences in *Myc* expression between the early and late season samples in all months except for November and February. This same general trend in *Myc* expression was also seen in the laboratory-maintained bees with significant difference in expression in all months except for November, February, and May. These results support previous conclusions from our research showing that the molecular regulation of diapause development in ALCB is not a simple singular cascade of gene expression but a highly plastic response that varies between bees depending upon their environmental history.

## Introduction

Diapause is a state of developmental dormancy that many insects undergo to survive changes in resource availability and below optimal temperatures in the winter months. Diapause can be divided into several ecophysiological phases: induction, preparation, initiation, maintenance, termination, and post-diapause quiescence, with finally resuming development (Kostal, 2006). These phases of diapause are regulated by both exogeneous and endogenous controls, including thermoperiod, photoperiod, and hormonal titers (Bell, 1968; Denlinger, 2002; Denlinger et al., 2005, 2012; Kostal, 2006; Sim and Denlinger, 2008, 2013). During diapause initiation, direct development ceases and is usually followed by metabolic suppression, processes which are both regulated by hormones (Denlinger et al., 2005, 2012), and possibly heat-shock proteins (Hayward et al., 2005; Rinehart et al., 2007). Although environmental conditions may still be favorable, diapausing individuals will maintain their course through endogenous controls, although environmental stimuli (ex. long-day photoperiod) may aid in diapause maintenance (Kostal, 2006; Kostal et al., 2017). The regulation of diapause termination is still not well understood but has been shown to be regulated by a combination of external and internal cues, such as chilling and change in photoperiod sensitivity, along with tissue sensitivity to stimuli (Kostal et al., 2000; Rinehart et al., 2001; Hodek, 2002). If environmental conditions are favorable, insects may resume direct development after diapause termination but most remain in post-diapause quiescence, a stage, that is, exogenously controlled by environmental conditions, such as day length and temperature (Kostal, 2006; Kostal et al., 2017). Finally, insects will resume direct development when they receive the appropriate environmental cues.

A key event in diapause is cell cycle arrest (Nakagaki et al., 1991; Denlinger, 2002; Denlinger et al., 2005, 2012; Kostal, 2006; Kostal et al., 2009, 2017; Hahn and Denlinger, 2011; Shimizu et al., 2018a,b). Cells are arrested at various points in the cell cycle by decreases in cell cycle regulators called cyclins (Vermeulen et al., 2003). Expression of these genes is controlled by several transcription factors, including proto-oncogene *Myc*, which activates transcription of cyclin substrates, cyclin-dependent kinases (CDK; Vermeulen et al., 2003). *Myc* is a downstream target of the Wnt/β-catenin pathway, a development-related signaling cascade (Chen and Xu, 2014). The Wnt/β-catenin pathway also interacts with targets of the insulin pathway, another pathway known to regulate insect diapause (Sim and Denlinger, 2008, 2013; Denlinger et al., 2012). Glycogen synthase kinase-3 beta-like (GSK-3β) is an antagonistic regulator of Wnt signaling and a downstream target of the insulin signaling pathway (IIS; Lin et al., 2009). A downstream target of Wnt/β-catenin signaling, β-catenin, interacts with Forkhead box protein O (FOXO), a downstream target of the insulin pathway that has been shown to be crucial for diapause regulation and stress responses (Sim and Denlinger, 2008; Sim et al., 2015). The insulin signaling pathway plays a role in creating diapause phenotypes through reduced metabolism (Hahn and Denlinger, 2011), enhanced stress tolerance (Wu and Brown, 2006; Sim and Denlinger, 2013; Matsunaga et al., 2016), and energy reserve accumulation (Satake et al., 1997). Previous studies showed that downregulation of the insulin signaling pathway plays a role in diapause regulation in both the mosquito *Culex pipiens*, and the alfalfa leafcutting bee (ALCB) *Megachile rotundata* (Sim and Denlinger, 2007, 2008; Cambron et al., 2021).

Our previous findings showed that ALCB overwintering conditions impacted the gene expression profile of bees that entered diapause early in the season, with temperature impacting expression levels of insulin pathway genes (Cambron et al., 2021). However, ALCB are a bivoltine species, with summer-emerging adults generating progeny (second generation) in the late summer that enter diapause in August/September (Yocum et al., 2018). This creates two diapause cohorts with the first entering diapause in June/July, and the second in the late summer, which begs the question of whether bees entering diapause later in the season also show similar expression profiles. Because temperature impacted IIS pathway gene expression in early season overwintering bees but late season bees are not exposed to summer temperatures, therefore expression levels for temperature-regulated genes, such as *Samui* and targets of the IIS pathway, may be differentially regulated. We hypothesize that expression profiles of bees entering diapause later in the season will differ from those in the early season. To better understand the effects of the timing of diapause initiation, we analyzed mRNA copy number of genes known to be involved in diapause regulation in early and late season diapausing ALCB.

## Materials and Methods

### Experimental Design

Overwintered *M. rotundata* samples were obtained from a previously published experiment (Yocum et al., 2018). In the summer of 2010 *M. rotundata* were reared as described in Yocum et al. (2018). Briefly, adults were released into an on-farm facility (Logan, UT, 41°47′37.04″N; 112°8′18.35″W). Nests that were removed from nesting blocks between the 30th of June and the 19th of July 2010 were first generation and designated as “early” season nests, and those removed on the 1st of September were second generation and designated “late” season nests. Both early and late offspring were placed into temperature treatment groups in October of 2010, consisting of 16°C for 2 weeks and then transferred to either a laboratory setting of 4–5°C and darkness (Constant), or left in the field shelter exposed to naturally fluctuating conditions (Field; Supplementary Figure S1). A HOBO Datalogger (Onset Computer Corp., Bourne, MA, United States) was used to record temperatures outside and inside the field shelter, showing that field samples were exposed to temperatures ranging from −18°C (January 2011) to 35°C (May 2011; Supplementary Figure S1; Yocum et al., 2018). Every month, individual bees were collected from both seasonal nests within each temperature treatment, flash-frozen in liquid nitrogen, and stored in −80°C until used in RNA extraction.

### Sample Preparation

RNA was extracted from frozen prepupae ground in liquid nitrogen and the resulting frozen powder was transferred to a 1.5 ml microcentrifuge containing TRIzol (Invitrogen, Life Technologies, Grand Island, NY, United States). The samples were extracted according to manufacturer’s instructions. RNA samples from all months (November, December, January, February, March, April, May, and June), early and late season, and temperature treatments were used. RNA pellets were prepared and quantified as previously described (Cambron et al., 2021). Four bees per treatment and seasonal group were used for nCounter analysis.

### nCounter Analysis

The same custom probe set from Cambron et al. (2021) was used for this study. Genes from the insulin pathway, downstream proto-oncogenes, and cell cycle regulators were measured (Supplementary Table S1; Cambron et al., 2021). RNA samples were prepared for nCounter analysis as previously described (Cambron et al., 2021), and shipped on dry ice to the University of Minnesota Genomics Center (Minneapolis, MN) for processing with nCounter Analysis System (NanoString Technologies Inc.). Resulting copy numbers were normalized to the geometric mean of the 10 reference genes used previously (Cambron et al., 2021). Normalized data set (Supplementary Table S2) is provided.

### Statistical Analysis

JMP Pro software (v.15.2.1, SAS Institute Inc., 2019, Cary, NC, United States) and SAS (v.9.4 SAS/STAT 15.1, SAS Institute Inc., 2018, Cary, NC, United States) were used for statistical analyses. Hierarchical cluster analysis with a Ward linkage method was performed with JMP Pro (v.15.2.1, SAS Institute Inc., 2018, Cary, NC, United States). To explore the relationship between season and temperature treatment, an additional variable was created (season + treatment, ST) since a season × temperature treatment interaction in our model would have only allowed for one *post hoc* comparison and an incomplete estimation of the effect due to month. The linear relationship between copy number (gene expression level) and the interaction of month and ST for each gene was modeled with a random coefficient growth curve model (RCGCM; Vonesh, 2012), and each gene’s regression line was determined as previously described (Cambron et al., 2021). *Post hoc* analyses were conducted by comparing upper and lower confidence limits between seasonal groups for each temperature treatment group across months. Limits that did not overlap were considered significantly different with 95% confidence. Copy number means +/− SEM and 95% CI are reported.

## Results

Of the 30 genes tested, 73% showed a significant interaction of month and ST, and 5% were significant only by season (Table 1). *Insulin-like growth factor I* was not significantly different by any effect (Table 1), and *AKTIP* and *PTEN* were unable to be fitted to the model.

**Table 1:** Fixed effects for each gene determined by nCounter analysis.

Gene	Fixed effect	ProbF
3-phosphoinositide-dependent protein kinase 1 (PDK1) (LOC100876379)	ST	2.10316E-26
(AKH) gonadotropin-releasing hormone II receptor (LOC100882851)	Month_Code*ST	5.67055E-23
Adipokinetic prohormone type 2-like	Month_Code*ST	0.004407442
Cyclin D	Month_Code*ST	0.006452544
Cyclin E	Month_Code*ST	1.80812E-19
Cyclin G	ST	2.5069E-70
Cyclin K	Month_Code*ST	0.023880333
Dual specificity mitogen-activated protein kinase kinase dSOR1 (ERK)	Month_Code*ST	1.86988E-09
Forkhead box protein O (LOC100882630)	Month_Code*ST	2.40219E-08
Glycogen synthase kinase-3 beta-like (LOC100882062)	Month_Code*ST	1.02862E-09
GRB2-associated-binding protein 2 (LOC100883689)	Month_Code*ST	3.07468E-05
GTP-binding protein Rheb homolog (RHEB) (LOC100880016)	ST	3.59785E-33
Insulin receptor substrate 1 (LOC100879231)	Month_Code*ST	5.95177E-10
Insulin receptor-like (LOC100879880)	Month_Code*ST	0.002568121
Insulin-like growth factor I (LOC100880980)	None	-
Insulin-like receptor (LOC100882691)	Month_Code*ST	3.28794E-12
Mitogen-activated protein kinase 1 (LOC100878492)	Month_Code*ST	1.06196E-10
Mitogen-activated protein kinase 14B-like (LOC100881316)	Month_Code*ST	3.31271E-06
Phosphatidylinositol 3-kinase catalytic subunit type 3 (LOC100882290)	ST	5.38029E-26
Phosphatidylinositol 3-kinase regulatory subunit alpha (p85) (LOC100878497)	Month_Code*ST	6.25041E-06
Protein L-Myc-1b (LOC100879259)	Month_Code*ST	0.003533487
Protein kinase C (LOC100879571)	Month_Code*ST	0.020039638
Protein son of sevenless (SOS) (LOC100883790)	ST	2.96538E-31
Ras-like protein 1 (LOC100876644)	Month_Code*ST	4.84856E-13
Ras-like protein 2 (LOC100879495)	Month_Code*ST	0.0019968
Samui	Month_Code*ST	2.74549E-05
Serine/threonine-protein kinase mTOR (LOC100880773)	Month_Code*ST	1.25965E-05
Tether containing UBX domain for GLUT4 (LOC100882950)	Month_Code*ST	8.6691E-11

*ST indicates season by temperature treatment variable, Month_Code *ST indicates the effect of season by temperature treatment over time.

### Expression Profiles Differed Between Early and Late Season

**Figure 1:**
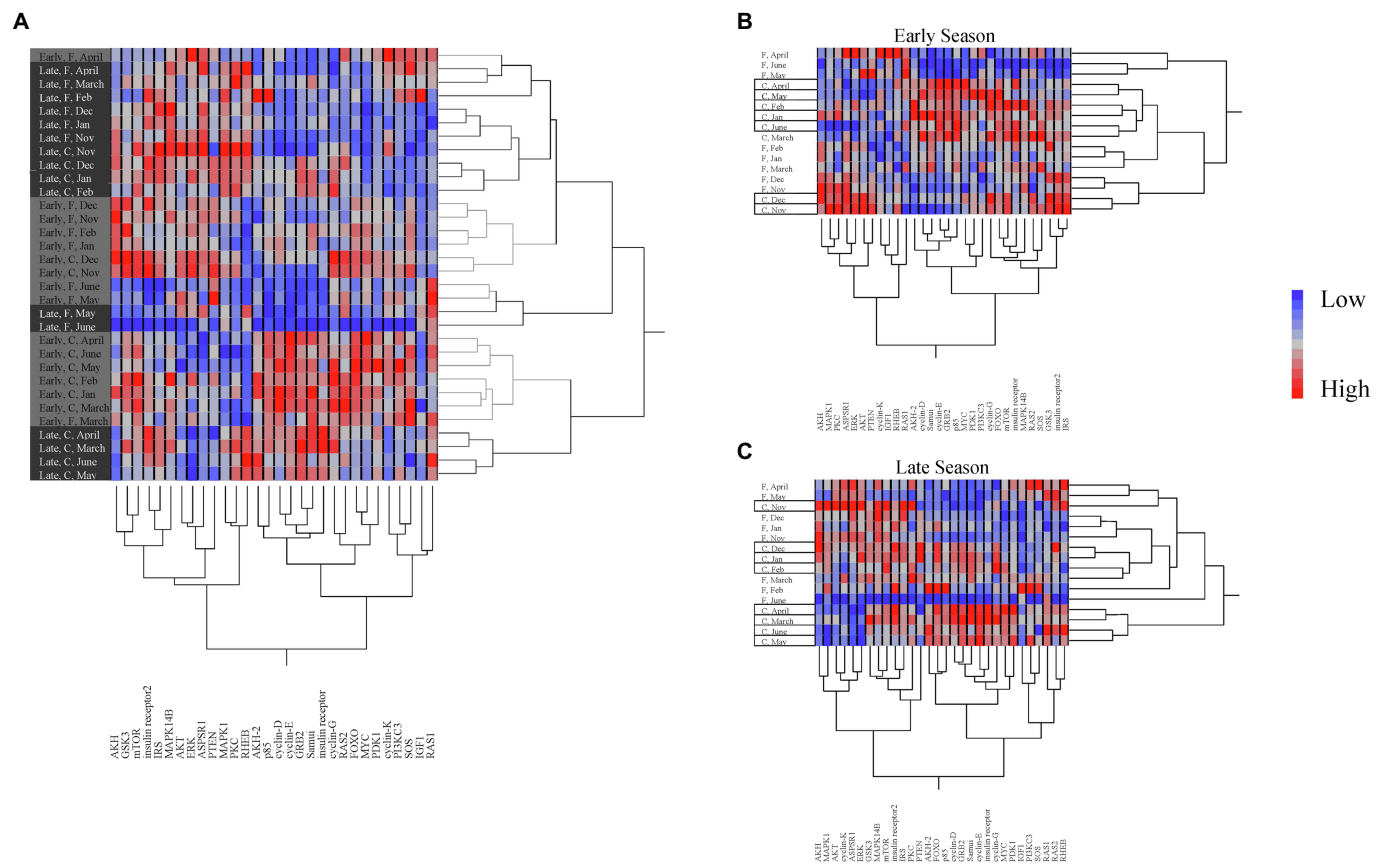
Two-way cluster analysis of gene expression showing **(A)** between seasons and **(B)** only Early season and **(C)** only Late season. Seasons are color-coded as light gray for Early season and dark gray for Late season. Temperature treatments are shown between seasons as field (F) and constant (C), and within seasons as field in open text and constant in boxed text. Expression gradient of blue to red indicates low to high expression, respectively.

When looking at overall expression profiles, two-way hierarchical cluster analysis showed different profiles for early and late season diapausers (Figure 1A). When comparing individual season profiles side by side, it was clearly shown that gene regulation is impacted by season as shown by distinctly different expression patterns (Figures 1B,C). Additionally, each season had different clustering of expression by months. In the early season, months grouped into two clusters for each temperature treatment groups, with the field temperature group dividing into (1) November to March and (2) April to June, while the constant temperature treatment group divided into (1) November to December, and (2) January to June (Figure 1B). For the late season, there were three clusters for each treatment group. Field temperature group broke up into (1) November to January, (2) February, March, June, and (3) April to May, while the constant temperature treatment group broke up into (1) November, (2) December to February, and (3) March to June (Figure 1C). Clustering of genes was also different by season, with early season branching from *Samui* and *cyclin E* (Figure 1B), but late season branching from *p85* and *FOXO* (Figure 1C).

### Insulin Pathway Targets Differently Expressed by Season

**Figure 2:**
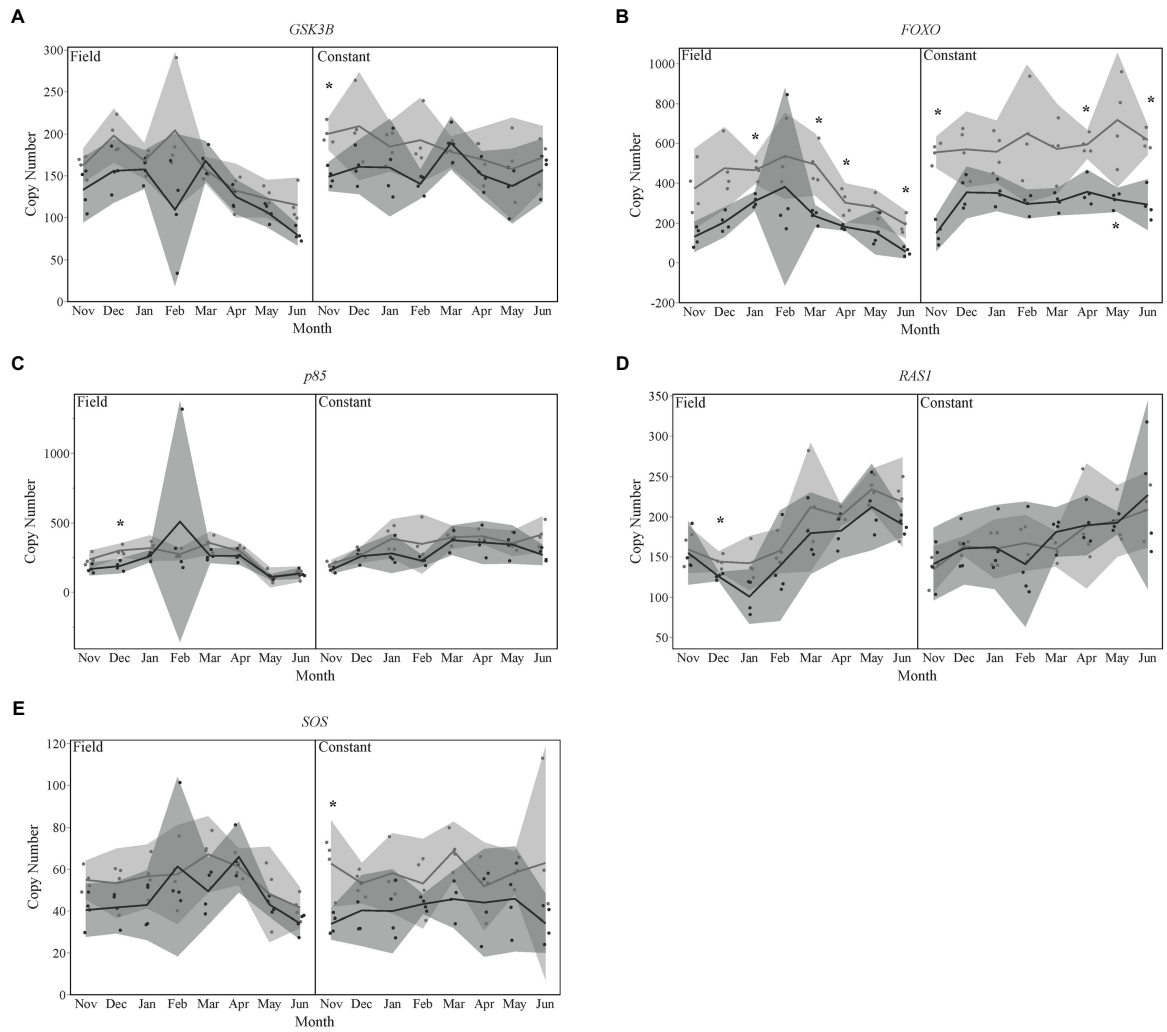
Gene expression for insulin signaling pathway targets **(A)** *GSK3β*, **(B)** *FOXO*, **(C)** *p85*, **(D)** *RAS1*, and **(E)** *SOS*. Seasons are color-coded as light gray for Early season and dark gray for Late season. Shaded areas indicate 95% CIs. Asterisks indicate months that were statistically significant between seasons.

Five downstream targets of the insulin signaling pathway exhibited significantly different expression by season. Levels of *GSK3β* were significantly lower in late season bees for the month of November in the constant temperature treatment group (Figure 2A). *FOXO* also showed lower levels of expression in late season diapausing bees for January, March, April, and June of the field temperature group and November, April, May, and June of the constant temperature treatment group (Figure 2B). December expression levels of *p85* were lower for late season diapausers overwintered in field temperatures (Figure 2C). *SOS* expression levels were significantly lower in November for late season diapausers that were overwintered in constant temperatures (Figure 2D). Lastly, expression levels for *RAS1* were also significantly lower in late season diapausers in December that were overwintered under field temperatures (Figure 2E).

### Seasonal Expression of Transcription Factors Differed

**Figure 3:**
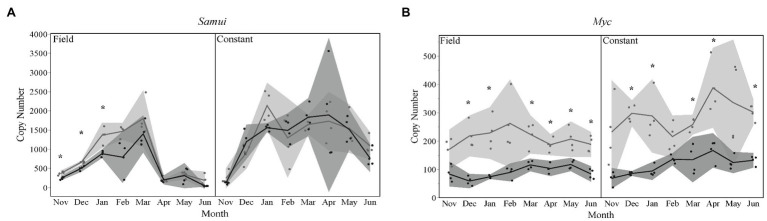
Gene expression levels for transcription factors **(A)** *Samui* and **(B)** *Myc* over time, for each temperature treatment. Seasons are color-coded as light gray for Early season and dark gray for Late season. Shaded areas indicate 95% CIs. Asterisks indicate months that were statistically significant between seasons.

For bees that were overwintered under field temperatures, those that entered diapause later in the season had significantly lower expression levels of *Samui* in the months of November through January (Figure 3A). Expression levels of *Myc* were significantly lower for late season of both temperature treatment groups for the months of December, January, March, April, May (field treatment only), and June (Figure 3B).

### Seasonal Regulation of Cell Cycle and Growth Genes

**Figure 4:**
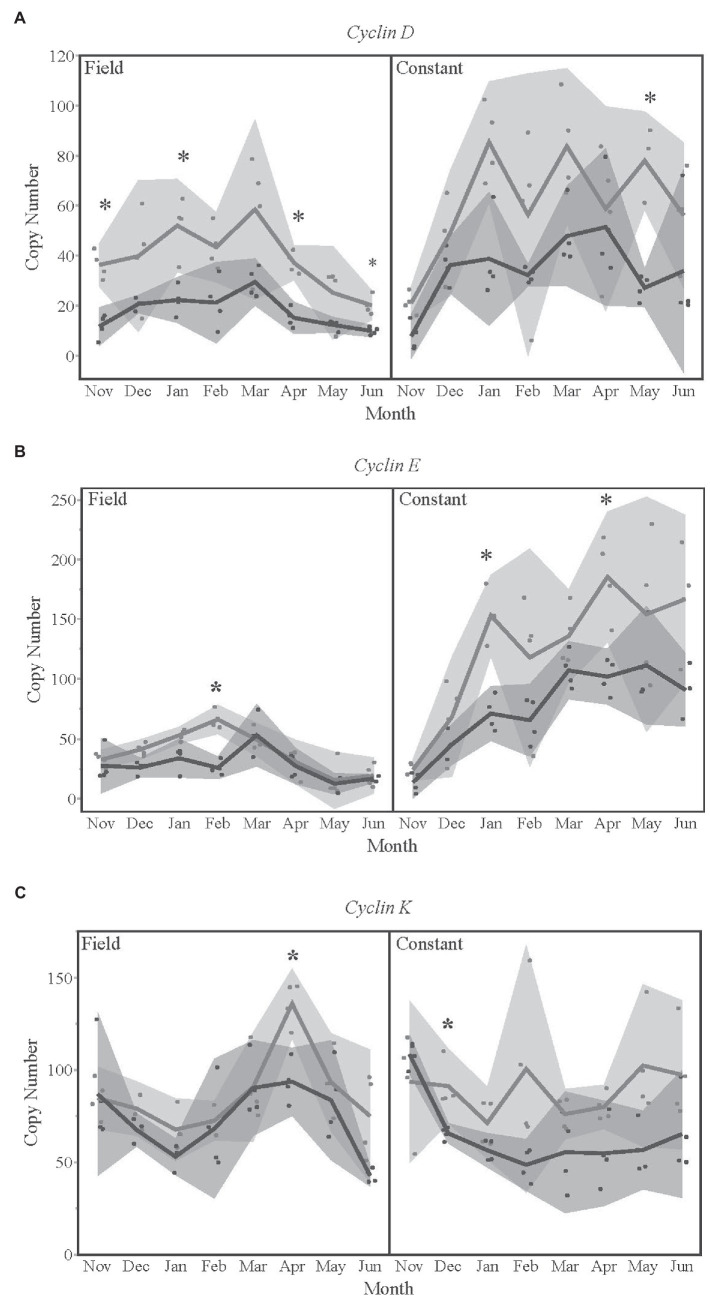
Gene expression levels for **(A)** *cyclin D*, **(B)** *cyclin E*, and **(C)** *cyclin K* over time, for each temperature treatment. Seasons are color-coded as light gray for Early season and dark gray for Late season. Shaded areas indicate 95% CIs. Asterisks indicate months that were statistically significant between seasons.

*Cyclins D*, *E*, and *K* were all significantly different by season for a given treatment temperature and month (Figure 4). The late season diapausers had significantly lower expression levels of *cyclin D* (Figure 4A) in November, January, April, and June of the field temperature group, as well as in May of the constant temperature treatment group. This pattern of late season having lower expression levels was also true for *cyclin E* (Figure 4B) and *cyclin K* (Figure 4C) for several months in both temperature treatment groups.

## Discussion

The objective of this study was to investigate the impact of the timing of diapause initiation and the overwintering environmental conditions on gene expression over the course of diapause development in the ALCB. Our results showed that the transcription profiles of bees at a specific time-point during winter depends on when the bee initiated diapause during summer (i.e., early or late) as well as the temperature conditions where it overwintered (i.e., field vs. constant). These differences in expression profiles included cell cycle regulator genes, transcription factors, and downstream targets of the IIS pathway. Although early season diapausing prepupae were already 1–2 months older than late season prepupae when we collected samples, we were surprised to find similar results to our previous findings with early season diapausing bees of only downstream targets and not the whole IIS pathway changing during overwintering (Figure 2). Two transcription factors, *Myc* and *Samui* were significantly different by season and over time (Figure 3), which made sense given their roles in regulating development. Our results demonstrated that at any given time point over the course of diapause development, gene expression profiles may vary between two individual bees due to timing of diapause initiation and their overwintering thermal history (Figure 1).

When comparing early and late season data together, the branching comes between *cyclin D* and *cyclin E* (Figure 1A). Cyclins have different functions throughout the cell cycle, with D types being present throughout actively dividing cells and E types only being present in cells that are in mid-G1 to mid-S phase (Lents and Baldassare, 2016; Shimizu et al., 2018b). Looking at overall expression profiles, we observed that gene expression in early season diapausing bees cluster into two groups by temperature treatment (Figure 1B), whereas late season diapausing bees showed more variation with grouping more similar to the ecophysiological phases of diapause (Figure 1C; Kostal, 2006). This clustering was originally seen in early season diapausing bees (Figure 2; Cambron et al., 2021), but we did not see the same pattern this time possibly due to needing a slightly different model to account for season. However, we did still see gene clusters main branching between *Samui* and *cyclin E* as shown previously (Cambron et al., 2021). Interestingly, the main branch for late season bees was between *FOXO* and *p85* (Figure 1C), both which regulate insulin signaling (Kops et al., 2002; Ueki et al., 2003; Luong et al., 2006; Sim and Denlinger, 2008; Grewal, 2009). These differences in branching may indicate that early and late season diapausing bees sampled at the same time points are physiologically different from each other.

Constant temperature exposures are not ecologically relevant to ALCB’s life history and have been shown to be detrimental. Exposing developing ALCB to constant temperatures results in the adults emerging randomly throughout the day and over more days than bees exposed to a fluctuating thermal regime (Yocum et al., 2016; Bennett et al., 2018). Whereas bees exposed to some form of fluctuating thermal regime display synchronous emergence around the beginning of the photophase and emerge over fewer days than bees under constant temperatures. Bees exposed to constant low temperatures during prepupal to adult development are less cold tolerant than bees exposed to some form of fluctuating thermal regime (Yocum et al., 2019). Finally, constant low-temperature exposure has been demonstrated to induce sublethal effects that negatively impact the bee fitness (Bennett et al., 2015). At the transcription level, bees exposed to constant temperature treatment display a different gene expression profile than bees exposed to field temperatures (Torson et al., 2015, 2017; Melicher et al., 2019; Cambron et al., 2021).

It would be expected that both temperature treatment groups of bees (constant vs. field) would experience different forms of stress, or degree of stress of a common stress, and therefore result in different gene expression patterns. Indeed, this was the case demonstrated in this investigation. Based on our previous results, we conclude that constant temperature exposure should be considered as a form of physiological stress. For example, *Samui* is a cold-induced gene, and in our study, both early and late season bees overwintered in field conditions increased expression levels with increasing cold exposure (Figure 3A). However, overwintering bees at a constant temperature in a laboratory setting led to expression levels of *Samui* to be constant with high variability (Figure 3A). Although *Samui* has been shown to be temperature-regulated (Moribe et al., 2010), the interaction of diapause regulation and temperature on *Samui* expression is still unclear.

Surprisingly, of all the differentially expressed genes, the proto-oncogene *Myc* was the most impacted by timing of diapause initiation. *Myc* plays a pivotal role in mitochondrial biogenesis (Li et al., 2005). In the cotton bollworm *Helicoverpa armigera*, hypoxia-inducible factor (HIF-1α) suppresses *Myc* activity, decreasing mitochondrial activity and leading to developmental arrest (Lin et al., 2016). Interestingly, there is another mechanism for diapause initiation involving *Myc*. In response to low levels of ecdysone, *Myc* downregulates the activity of hexokinase, a gene important for insect development and metabolic activity (Lin and Xu, 2016). In our study, *Myc* expression levels were higher in early season diapausing bees, regardless of temperature treatment (Figure 3B). The month of February showed several significant differences between seasons and even within seasons, especially for *Myc*. Upon further investigation of our temperature data (Supplementary Table S3), the month of February showed a peak in maximum daily temperatures, therefore increasing daily mean temperatures and potentially causing drastic changes in gene expression. For *Myc*, the month of February was not significantly different due to such high variability (Figure 2B). Future studies would need to also include ecdysone and hexokinase to investigate the differences in *Myc* expression between seasons. Our study highlights the role of *Myc* in seasonal impacts on expression profiles, but more studies will be needed to understand the mechanism behind the role of *Myc* in diapause regulation between seasons, and whether it impacts thermal tolerance.

*Myc*, *FOXO*, and cyclin genes were the most differentially expressed genes between early and late season diapausing bees (Supplementary Table S1). The majority of genes impacted by the timing of diapause were not in the IIS pathway but rather regulate it or regulate development. This provides further support showing that the IIS pathway does not change during overwintering and that the IIS pathway suppression is a key process in diapause regulation (Denlinger, 2002; Sim and Denlinger, 2008, 2009, 2013). However, diapause is a complex network of regulatory processes that can vary drastically depending on the environmental conditions experienced prior to and during diapause, and many more factors that remain insufficiently investigated.

Our investigation demonstrated that at the time points, we measured the early and late season bees were transcriptionally different as determined by differential gene expression patterns. Obvious questions arise from these results, such as (1) what is the mechanism driving these differences, (2) can the genes found to be differentially regulated over the course of diapause development be subdivided into those primarily regulated by environmental factors and those that directly regulate diapause development, (3) how or do these two groups of genes interact to shape the diapause phenotype, and (4) are these differences biologically significant? One possible mechanism for the differences, we found is that the bees are at different points in their diapause development. Therefore, the early season bees are further along in their diapause development than the late season bees. As attractive as this explanation is, it has been demonstrated in other insect species that the timing of diapause initiation regulates the duration of diapause (Danks, 1987). Entering diapause later in the season resulted in a shorter diapause thereby aiding in synchronizing the post-diapause emerging insects. To start to resolve these questions in future investigations, we will need well-defined physiological landmarks to ensure that the insects being compared are at the same point in their diapause development. For an exemplary example of using physiological landmarks to investigate the molecular regulation of diapause, see Ragland et al. (2011).

## Data Availability Statement

The gene expression datasets generated for this study can be found on Dryad (doi 10.5061/dryad.h9w0vt4kb).

## Author Contributions

LC-K, GY, and KG designed the experiment and involved in funding. LC-K collected data, wrote first draft of the manuscript, and created all figures. LC-K, GY, and KY conducted statistical analysis. KG, GY, and KY edited the manuscript. All authors contributed to the article and approved the submitted version.

## Funding

This research was funded by a National Science Foundation Graduate Research Fellowship and a Graduate Research Internship with the USDA-ARS Fargo, ND, to LC-K, an NSF EPSCoR-1826834 to KG, and USDA-ARS funding 3060-21220-032-00D to GY.

## Conflict of Interest

The authors declare that the research was conducted in the absence of any commercial or financial relationships that could be construed as a potential conflict of interest.

## Publisher’s Note

All claims expressed in this article are solely those of the authors and do not necessarily represent those of their affiliated organizations, or those of the publisher, the editors and the reviewers. Any product that may be evaluated in this article, or claim that may be made by its manufacturer, is not guaranteed or endorsed by the publisher.
