# Supplementary material for: Timing of Diapause Initiation and Overwintering Conditions Alter Gene Expression Profiles in Megachile rotundata
Source: Front Physiol. 2022 Mar 8;13:844820. doi: 10.3389/fphys.2022.844820 (PMC8957994; doi:10.3389/fphys.2022.844820)
Supplement: Supplementary file 1 [file Image_1.PDF]

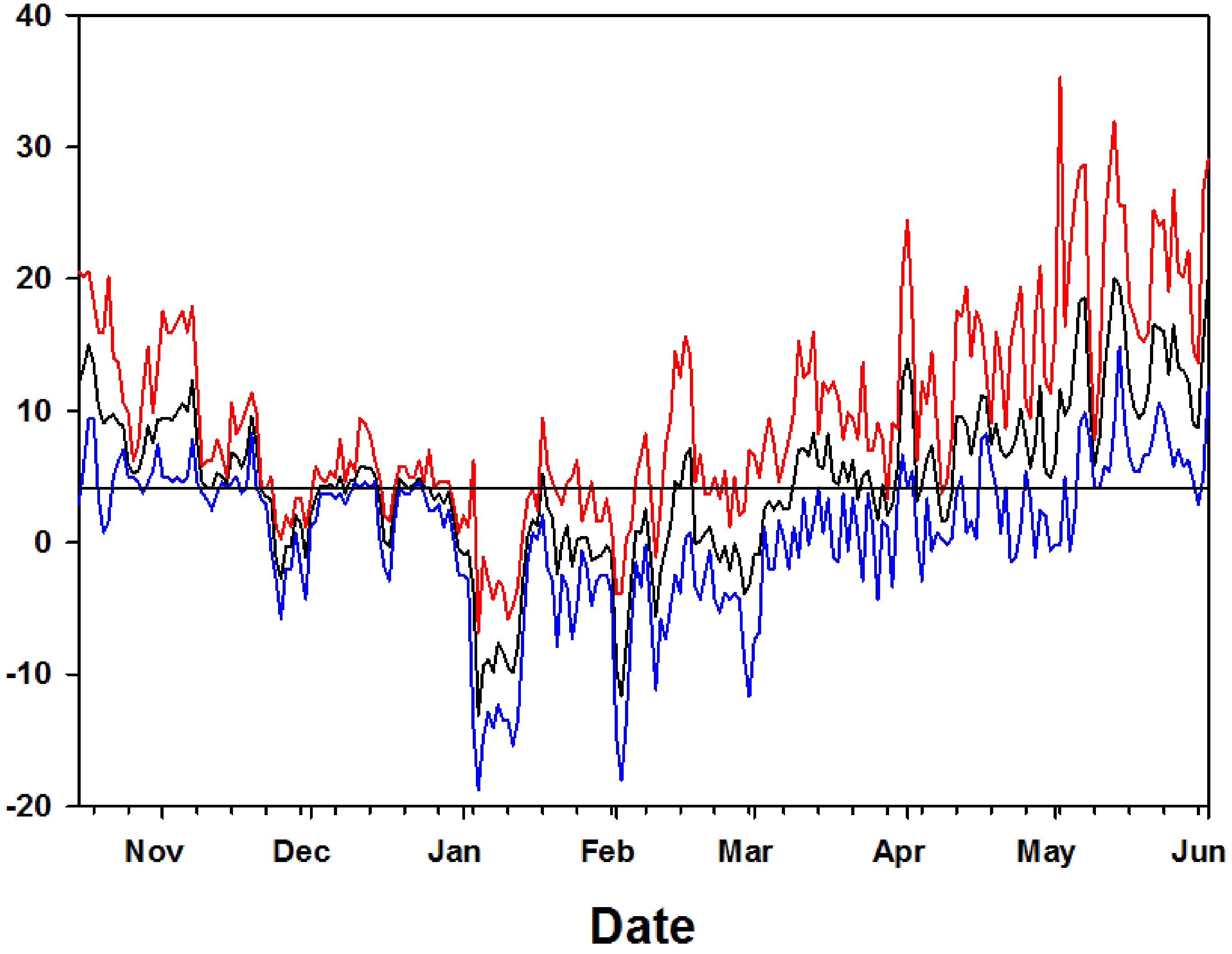

Figure S1. Field mean (black), minimum (blue), and maximum (red) temperatures experiences by diapausing prepupae left outdoors after October 22, 2010. Constant temperature treatment of 4-5˚C s hown by black line. Figure modified from Fig. S1 in Yocum et al. 2018.
