# Supplementary material for: Timing of Diapause Initiation and Overwintering Conditions Alter Gene Expression Profiles in Megachile rotundata
Source: Front Physiol. 2022 Mar 8;13:844820. doi: 10.3389/fphys.2022.844820 (PMC8957994; doi:10.3389/fphys.2022.844820)
Supplement: Supplementary file 2 [file Table_1.docx]

# Supplementary Materials

Table S1. Summary statistics for genes with significantly different expression levels between seasons for a given month by temperature treatment as determined by nCounter analysis. Summary statistics include lower and upper limits for 95% confidence intervals for Early and Late seasons. Confidence intervals that did not overlap were considered statistically significant.

| Gene | Month | Treatment | Lower_Early | Upper_Early | Lower_Late | Upper_Late |
| --- | --- | --- | --- | --- | --- | --- |
| cyclin-D | Nov | Field | 27.68644118 | 45.08355882 | 4.126995239 | 19.40800476 |
|  | Jan | Field | 33.46742606 | 70.72257394 | 13.23326384 | 31.43673616 |
|  | Apr | Field | 30.33014118 | 44.23985882 | 8.900907003 | 21.554093 |
|  | Jun | Field | 14.3026882 | 26.1623118 | 7.4971028 | 12.3728972 |
|  | May | Constant | 58.47597729 | 97.68902271 | 19.55733605 | 34.87266395 |
| cyclin-E | Feb | Field | 53.99398674 | 78.28101326 | 16.32551797 | 34.96948203 |
|  | Jan | Constant | 119.137332 | 187.182668 | 48.25525976 | 93.97974024 |
|  | Apr | Constant | 130.9170561 | 240.1279439 | 78.77064607 | 125.2893539 |
| cyclin-K | Apr | Field | 116.9781008 | 154.7168992 | 75.24001505 | 111.949985 |
|  | Dec | Constant | 71.22734882 | 111.2376512 | 60.18207526 | 71.05292474 |
| FOXO | Jan | Field | 396.4375404 | 534.2624596 | 263.7963669 | 357.3636331 |
|  | Mar | Field | 338.898698 | 649.546302 | 179.6350036 | 289.7049964 |
|  | Apr | Field | 204.3164051 | 399.8035949 | 161.7553166 | 199.5596834 |
|  | Jun | Field | 124.0740372 | 262.2009628 | 22.44720188 | 90.59279812 |
|  | Nov | Constant | 476.6042507 | 635.6107493 | 60.8220671 | 239.8029329 |
|  | Apr | Constant | 523.6581674 | 666.9018326 | 246.5190383 | 468.0059617 |
|  | May | Constant | 380.4522103 | 1055.54279 | 256.6889095 | 377.2610905 |
|  | Jun | Constant | 543.3360048 | 691.6589952 | 166.1275562 | 420.0424438 |
| GSK3 | Nov | Constant | 181.0087765 | 219.6412235 | 131.911351 | 166.343649 |
| MYC | Dec | Field | 146.4727769 | 290.2172231 | 33.88914434 | 84.35085566 |
|  | Jan | Field | 137.9935007 | 319.2414993 | 66.65381265 | 81.83118735 |
|  | Mar | Field | 156.2850647 | 291.4149353 | 93.85469637 | 138.6503036 |
|  | Apr | Field | 150.3477854 | 224.0572146 | 76.83586007 | 130.5441399 |
|  | May | Field | 144.7863426 | 269.0986574 | 94.36466129 | 141.0103387 |
|  | Jun | Field | 144.7585888 | 231.9864112 | 57.02756969 | 111.3674303 |
|  | Dec | Constant | 252.6479338 | 343.7920662 | 77.57828884 | 94.44171116 |
|  | Jan | Constant | 159.8858978 | 418.0991022 | 63.17914606 | 126.3008539 |
|  | Mar | Constant | 223.1654843 | 293.1695157 | 55.03359454 | 214.5064055 |
|  | Apr | Constant | 247.6092054 | 529.0657946 | 106.6435131 | 227.9014869 |
|  | Jun | Constant | 257.4815531 | 346.1084469 | 106.7971635 | 158.5128365 |
| p85 | Dec | Field | 252.3329558 | 351.1720442 | 136.6444002 | 242.3205998 |
| RAS1 | Dec | Field | 131.8766056 | 156.8783944 | 120.3227365 | 131.4172635 |
| Samui | Nov | Field | 300.8590748 | 441.4209252 | 186.9139061 | 297.8960939 |
|  | Dec | Field | 630.2617475 | 698.9682525 | 415.477005 | 593.692995 |
|  | Jan | Field | 1036.400863 | 1690.459137 | 773.9138458 | 997.1061542 |
| SOS | Nov | Constant | 42.03449218 | 83.17050782 | 26.40947047 | 41.48052953 |
